# Growth Suppression of a Gingivitis and Skin Pathogen *Cutibacterium* (*Propionibacterium*) *acnes* by Medicinal Plant Extracts

**DOI:** 10.3390/antibiotics10091092

**Published:** 2021-09-09

**Authors:** Hyoung-An Choi, Sang-Oh Ahn, Ho-Dong Lim, Geun-Joong Kim

**Affiliations:** Department of Biological Sciences, College of Natural Sciences, Chonnam National University, 77 Yongbong-ro, Buk-gu, Gwangju 61186, Korea; hyoungan45@kakao.com (H.-A.C.); repaul2001@gmail.com (S.-O.A.); eastlake@cialm.or.kr (H.-D.L.)

**Keywords:** *Propionibacterium acnes*, *Cutibacterium acnes*, plant extract, antimicrobial activity

## Abstract

*Propionibacterium acnes*, newly reclassified as *Cutibacterium acnes*, is an anaerobic Gram-positive bacterium causing acne, found mainly on the skin. In addition, *P. acnes* is responsible for inflammation of the gums (gingivitis) and blood vessels, consequently leading to various diseases in the human body. In recent years, the evolution of microorganisms, such as *P. acnes,* that have become resistant to many commercial antibiotics due to the widespread use of antimicrobial drugs in the treatment of infectious diseases has emerged as a major clinical problem. We here analyzed the potential use of 37 medicinal plant extracts as plausible candidates for treating *P. acnes*, in terms of total phenolic and flavonoid contents, antioxidants scavenging and antimicrobial activity. Consequently, methanol extracts from 14 medicinal plants showed promising antimicrobial activities against *P*. *acnes*. In particular, as the extracts from *Chrysosplenium flagelliferum* F. and *Thuja orientalis* L. exhibited distinct antimicrobial activities in both the broth dilution and disc diffusion assay, they could be effectively used as active ingredients for preventing or treating inflammatory periodontal diseases, such as periodontitis.

## 1. Introduction

*Propionibacterium acnes* (newly reclassified as *Cutibacterium acnes*) is a non-spore-forming anaerobic Gram-positive bacterium frequently found in the sebaceous glands of the human body. It is also a causative organism closely linked to various inflammatory diseases by infecting other organs, including the skin, airways, heart valves, eyes, gums, and others [1,2,3,4]. A representative example of these inflammatory diseases is acne on the skin, and other such diseases associated with *P. acnes* range from those occurring in artificial joints and oral prostheses to more severe diseases occurring in the heart valve areas [5,6]. In a certain condition, *P. acnes* confers an increased bioadhesive properties of biofilms formed around prostheses [5]. Inflammation caused by *P. acnes* in the oral cavity also causes gum edema and bleeding. Topical and orally administered systemic agents have been used to treat inflammation caused by *P. acnes* [7]. However, currently used drugs, including erythromycin [2], isotretinoin [8], benzoyl peroxide [9], triclosan [10,11], tetracycline, and azelaic acid [2,9], have side effects, such as cheilitis, xerostomia, teratogenesis, and thrombosis, induce antibiotic resistance with long-term use, and retain a high chance of relapse of the disease upon discontinuation of use [2,8]. In particular, antibiotic resistance is considered a serious problem affecting common bacterial strains in the human body [8,12]. In addition, according to a biofilm study involving *P. acnes*, the species acts as a bioadhesive and is involved in the formation of comedones or acts on the male hormone androgen or insulin-like growth factors [5,6].

To identify alternative solutions to the adverse effects and antibiotic resistance caused by the extensive and indiscriminate use of current commercial antibiotics, much research has been actively conducted to confirm the antimicrobial activities of various plant extracts in many research fields. In particular, according to some recent studies, hop (*Humulus lupu-lus* L.)-CO2-extract [13]; ethanol extract of oregano (*Origanum vulgare* L.) [14]; ethanol extract of cassava leaves (*Manihot esculenta* C.) [15]; ethanol extract of cinnamon bark (*Cinnamomum cassia* J., *C. zeylanica* and *C. loureirii*) [16]; methanol extracts of twenty-five Egyptian plants, namely *Myrtus communis* L., *Curcuma longa* L., and *Myristica fragrans* H. [17]; methanol seed extract of *Tamarindus indica* L. and *Syzygium cumini* L. [18]; 70% ethanol extracts of propolis [19]; cold water, hot water, and methanol extracts of *Sanguisorba officinalis* L. root [20]; and deionized water, 80% ethanol, and 100% methanol extracts of ten *Lauraceae* species [21] have antimicrobial properties against *P. acnes*.

In this study, 37 oriental medicinal plants that have been used for treating inflammatory and skin diseases in Korea were selected based on information in the literature, such as folk remedies and herbal medicine publications, and antimicrobial abilities of their methanol extracts against *P. acnes* were explored. This study provides basic data for overcoming the limitations of conventional drugs in treating inflammation caused by *P. acnes* and developing novel anti-inflammatory therapeutic substances.

## 2. Results and Discussion

### 2.1. Total Phenolic and Flavonoid Contents

As is well known, the various beneficial effects of plant extracts on human physiology are closely correlated with their secondary metabolite contents. In particular, phenolic compounds in spices and herbal ingredients are representative antioxidant substances [22]. The degree of their antioxidant activity greatly depends on the types of plants and their active ingredients, and the methods of extracting these active ingredients from the plants [23]. Therefore, the phenolic contents in the methanol extracts were determined, and the antioxidant activity was then analyzed. The phenolic contents in the methanol extracts of 37 plant species were determined following the Folin–Denis method, as described in the Methods and Materials Section and shown in Table 1.

Table 1 shows the total phenolic contents in the plant extracts used in this study, with the content of each methanol extract from the 37 medicinal plants calculated using the antioxidant value of gallic acid as a standard compound. As shown in Table 1, the highest total phenolic content was observed in *Hovenia dulcis* T. extract (197.12 μg/mL), and relatively high total phenolic contents were also observed in the extracts of *C. japonica* L. (150.47 μg/mL), *S. stoloniferum* B. (150.16 μg/mL), *P*. var. *lilacina* L. (195.96 μg/mL), *C. coreana* U. (150.01 μg/mL), *P. scabiosaefolia* F. (171.30 μg/mL), and *T. orientalis* L. (196.99 μg/mL).

Flavonoid compounds are various nitrogen-free biological pigments with a flavone backbone as the basic structure, and representative examples include anthocyanin and anthoxanthin. Flavonoid compounds are closely related to melanin production relaxation, hemostasis, and anti-inflammatory action [24,25]. In addition, flavonoid compounds, such as phenolic compounds, have been reported as representative substances with antioxidant and antimicrobial effects among active herbal ingredients, and the degree of their antioxidant and antimicrobial activities depends on the type of plants, plant parts used (stems, leaves, roots, and so on), type and proportion of their active ingredients, and active ingredient extraction methods [24]. To access these useful resources, the flavonoid contents in methanol extracts from 37 plant species were determined following the Nieva Moreno method, as described in the Method and Materials Section, and are also summarized in Table 1.

As shown in Table 1, the highest total flavonoid content was detected in *T. orientalis* L. extract (87.99 μg/mL), followed by *H. dulcis* T. (62.12 μg/mL), *I. integra* T. (57.84 μg/mL), *C. siderosticta* H. (53.71 μg/mL), *P. scabiosaefolia* F. (51.92 μg/mL), *M. azedarach* var. *japonica* M. (47.04 μg/mL), *C. flagelliferum* F. (39.74 μg/mL), and *C. keiskei* M. (37.81 μg/mL). Ahn et al. [26] obtained separate extracts from the leaves and fruits of *T. orientalis* L. using several extraction methods and determined the total phenolic and flavonoid contents of the extracts. The leaf–methanol extract contained 16.02% and 0.25% of phenolic and flavonoid contents, respectively, and the fruit–methanol extract contained 12.67% of phenolic contents and 0.09% of flavonoid contents. Additionally, the methanol extracts exhibited the highest total phenolic and flavonoid contents as compared to the water and ethanol extracts of both leaves and fruits. Meanwhile, Jung et al. [27] reported total phenolic and flavonoid contents of 14.43 and 15.69 mg/g in an ethanol extract of *C. majus* var. *asiaticum* H., and the total flavonoid content was 1.09-fold higher than the total phenolic content. These results are contrary to the results of this study, which showed that the total phenolic content was higher than the total flavonoid content. This difference may be due to differences in the arable land of *C. majus* var. *asiaticum* H. and the extraction method.

### 2.2. Antioxidant Activity of Plant Extracts

1,1-Diphenyl-2-picrylhydrazyl (DPPH) is a relatively stable free radical and a scavenger for other radicals. DPPH has a deep-violet color in solution and is decolorized by reduction with antioxidants and aromatic amines. These properties allow for the screening of antioxidants from various natural materials or the determination of their antioxidant activities. The antioxidant activity of plant extracts also depends on various characteristics, such as the soil properties, climatic change, and surrounding tree species in their habitat. To determine antioxidant activity, a DPPH assay was conducted using methanol extracts (1 mg/mL, 100 µL) from 37 medicinal plant species, and the results are shown in Table 2.

As shown in Table 2, when 0.1 mg of the plant extract was added to the final concentration of 1 mg/mL, the highest antioxidant activity was observed for *T. orientalis* L. (96.76%), followed by *G. sibiricum* L. (92.91%) and *C. japonica* L. (92.79%), while antioxidant activity above 80% was also observed in *I. integra* T., *S. stoloniferum* B., *L. glauca* S. and Z., *O. inularis* K., *A. elata* A., and *C. asiatica* L. Consistent with this result, Ahn et al. [26] reported that methanol-treated leaf and fruit extracts from *T. orientalis* L. exhibited antioxidant activity of 89.10% and 94.50%, respectively, although some fluctuations were possible due to the different solvents used in extraction. For most of the plant extracts exhibiting antioxidant activity above 80%, the total phenolic compound content was high. However, both *C. asiatica* L. and *Aralia elata* A. exhibited high antioxidant activity, despite their low phenolic compound contents.

### 2.3. Antimicrobial Activity of Plant Extracts

As *P. acnes* is a causative agent of various inflammatory diseases, screening substances with antimicrobial activity against *P. acnes* can provide a basis for the development of various drugs, including antimicrobial and anti-inflammatory agents. Therefore, we attempted to screen plant extracts with high antimicrobial activity against *P. acnes* from 37 plant species by analyzing the growth inhibition of *P. acnes* in a liquid culture with methanol extracts. Table 3 shows the antimicrobial activities of the methanol extracts of 37 medicinal plants.

As shown in Table 3, the extracts of *I. integra* T. (93%), *C. flagelliferum* F. (93%), *P. sagittatum* var. *sieboldii* L. (91%), *S. stoloniferum* B. (97%), *L. glauca* S. and Z. (91%), *C. siderosticta* H. (90%), *T. orientalis* L. (94%), and *C. japonica* L. (96%) exhibited very high (≥90%) antimicrobail activities against *P. acnes*. In addition, *R. rugosa* T., *H. rhombea* S. and Z., and *P. vulgaris* var. *lilacina* L. exhibited relatively high (≥80%) antimicrobial activities. In contrast, 12 plant extracts did not show any inhibitory effect on cell growth under defined conditions. There was no distinct relationship between growth inhibition and total phenolic and/or flavonoid contents. Subsequently, a disc diffusion assay was also conducted using 14 plant extracts with antimicrobial activities of over 70% for further investigation. The 14 selected plants were *I. integra* T., *C. japonica* L., *C. flagelliferum* F., *P. sagittatum* var. *sieboldii* L., *S. stoloniferum* B., *P. vulgaris* var. *lilacina* L., *L. glauca* S. and Z., *R. pseudo-accacia* L., *C. siderosticta* H., *G. sibiricum* L., *T. orientalis* L., *H. rhombea* S. and Z., *A. continentalis* K., and *R. rugosa* T. The resulting inhibition results are shown in Table 4.

As shown in Table 4, all 14 plant extracts exhibited antimicrobial activity at a concentration of 1 mg/disc, and the extract from *A. continentalis* K. showed high antimicrobial activity, even at a lower concentration of 0.1 mg/disc. Additionally, *C. flagelliferum* F., *C. siderosticta* H., *G. sibiricum* L., and *T. orientalis* L. showed relatively high antimicrobial activities at a concentration of 1 mg/disc. Therefore, the concentration range of the 14 plant extracts that showed antimicrobial activity was 0.1–1 mg/disc. The maximum antimicrobial activity was predicted to be observed at the concentration of 1 mg/disc, because, excluding *H. rhombea* S. and Z., similar antimicrobial activities were detected at concentrations of both 1 and 2 mg/disc. That is, the maximum antimicrobial activity was observed at a certain concentration and did not increase proportionally as the concentration of the plant extract increased. Sohn et al. [7] reported that extracts from *Chloranthus japonicus* S. Z., Sinomenium acutum T., *Sophora flavescens* A., *Evodia officinalis*, *Ginkgo biloba* L., *Mori cortex*, and *A. continentalis* K. have excellent antimicrobial activity against *P. acnes*, and that Kurarinone ((2*S*)-2-(2,4-Dihydroxyphenyl)-2,3-dihydro-7-hydroxy-5-methoxy-8-[(2*R*)-5-methyl-2-(1-methylethenyl)-4-hexen-1-yl]-4*H*-1-benzopyran-4-one) and Kuraridin ((*E*)-1-[2,4-dihydroxy-6-methoxy-3-(5-methyl-2-prop-1-en-2-ylhex-4-enyl)phenyl]-3-(2,4-dihydroxyphenyl)prop-2-en-1-one) were the principal active antimicrobial substances isolated from the *A. continentalis* K. extract. Choi et al. [2] reported that 55 medicinal plants, including *Terminalia chebula* R. and *Angelica reflexa* L., have antimicrobial activity against *P. acnes* and that the degree of antimicrobial activity varies depending on the medicinal plant species and type of extraction solvent. The highest amount of active antimicrobial substance can be obtained when ethanol is used as an extraction solvent. In their study, methanol extracts were used for comparison, and the antimicrobial activity differed between ethanol and methanol extracts.

Kim et al. [19] reported that 70% ethanol extracts from propolises collected from 10 regions of the world, including Korea, had antimicrobial activity against *P. acnes*. The antimicrobial activity of propolis is closely linked to its phenolic compounds, which are similar to those from typical plant extracts. However, the antimicrobial effect of propolis varies depending on its native habitat and extraction method. In the study, they used 70% ethanol extract from propolis for an antimicrobial assay, as a relatively high flavonoid content was extracted. Cho et al. [21] also reported that 80% ethanol extract from Lauraceae evergreen broad-leaved species (*Laurus nobilis* L.) exhibited antimicrobial activity against *P. acnes*, and used deionized water and 100% methanol as controls for activity validation.

Water [20,21], ethanol [2,14,15,16,19,21,27], methanol [17,18,20], and other chemicals [13] have been considered as extraction solvents for plant ingredients. However, the antimicrobial activity of the plant extract against *P. acnes* not only depends on the extraction solvent, but also on various factors, such as the plant part, extraction time, and temperature. These characteristics provide different results when conducting comparative analyses using reported data.

*P. acnes* is a common species throughout the human body that causes inflammation- related diseases, such as mild, moderate, and severe acne; chronic endophthalmitis; and inflammation caused by the implantation of artificial aids [2,28,29,30]. Among various natural habitats, the oral cavity is an optimal site for pathogenic microorganisms to invade or inhabit, and many pathogenic microorganisms enter the body through the oral cavity and cause diseases [31,32,33,34]. In this study, as a plausible alternative solution to the issue of antibiotic abuse and the resulting emergence of resistant bacteria, we attempted to determine the potential capability of the plant extracts to effectively control *P. acnes*. We verified that methanol extracts from 14 plant extracts, including *I. integra* T., *C. japonica* L., *C. flagelliferum* F., *P. sagittatum* var. *sieboldii* L., *S. stoloniferum* B., *P. vulgaris* var. *lilacina* L., *L. glauca* S. and Z., *R. pseudo-accacia* L., *C. siderosticta* H., *G. sibiricum* L., *T. orientalis* L., *H. rhombea* S. and Z., *A. continentalis* K., and *R. rugosa* T., had promising antimicrobial activities. While many studies have been conducted on *P. acnes* for preventing or treating skin diseases, studies on other diseases are rarely conducted. In particular, the mechanism by which *P. acnes* causes oral disease has not received much attention. *P. acnes* is a causative pathogen of edema and bleeding in the gums and increases the bioadhesiveness of biofilms formed around prostheses [5]. However, it has received less attention than typical oral pathogens, such as *Streptococcus mutans*. Although this study suggests the potential use of methanol extracts from 14 medicinal plants as suppressive agents for *P. acnes*, we did not further identify or isolate active antimicrobial ingredients in plant extracts. However, along with their antimicrobial activity, the free radical scavenging activity and total phenolic and flavonoid contents of the 14 plant extracts could provide insight into how they inhibit the cell growth of *P. acnes*.

In conclusion, the results of this study indicate that methanol extracts from 14 medicinal plants have promising antimicrobial activity against *P. acnes*. The data provide evidence that extracts from some medicinal plants could be used as basic materials for identifying substances that can prevent or treat skin and oral diseases, such as tooth decay and stomatitis, and be used as oral hygiene products, such as mouthwashes, toothpaste, varnishes, or the like. In particular, as the extracts from *C. flagelliferum* F. and *T. orientalis* L. exhibited excellent antimicrobial activities in both the broth dilution and disc diffusion assay, they could be effectively used as active ingredients for preventing or treating inflammatory periodontal diseases, such as periodontitis.

## 3. Materials and Methods

### 3.1. Medicinal Plant Materials

Medicinal plants in Korea were selected based on their antibiotic activity and their traditionally known medical uses. Dried whole-plant materials of *Platycladus orientalis*, *Camellia japonica*, and *Sparganium erectum* (600 g of each sample) were purchased from the Gyeong-dong (Korea) oriental medicine market, while freeze-dried methanol extracts of other plants (approximately 20 mg of each sample) were obtained from the Korea Plant Extract Bank. The medicinal plants used in this study are listed in Table 5.

### 3.2. Bacterial Strains and Culture Conditions

*P. acnes* 5527, a typical skin and gingivitis pathogen, was selected as a candidate to evaluate the antimicrobial activity of the plant extracts and was purchased from the Korean Collection for Type Cultures (KCTC) in the Korea Research Institute of Bioscience and Biotechnology (KRIBB, Daejeon, Korea). *P. acnes* was grown at 37 °C for 72 h in YPD (1% yeast extract, 2% peptone, and 2% dextrose) broth using an anaerobic culture kit consisting of an anaerobic jar and Anaerocult^®^ A (Merck, Darmstadt, Germany). The presence or absence of oxygen was confirmed using an anaerotest (Merck, Darmstadt, Germany).

### 3.3. Methanol Extraction of Plants

The dried ground raw materials of *T. orientalis* L., *C. japonica* L., and *S. stoloniferum* B. (50 g) were extracted three times with 1.3 L of 95% methanol for 3 d at room temperature (25 °C). Thereafter, the extracts were centrifuged at 8000× *g* for 30 min. The combined supernatants were evaporated using a rotary vacuum evaporator (IKA^®^ RV 10 Digital, Germany). The freeze-dried solids (approximately 20 mg) were dissolved in 1 mL of methanol to assess the antimicrobial activities.

### 3.4. Determination of Antimicrobial Activity

The cells of *P. acnes* KCTC 5527, prepared to determine the antimicrobial activity of plant extracts, were typically cultured in a YPD solid medium, selected as a single colony, and then inoculated in 10 mL of liquid medium. The antimicrobial activity of the plant extracts was primarily determined following the broth dilution method according to a previous report [16]. To this end, 30 µL of the passaged culture medium was inoculated in 3 mL of liquid medium, and each of the methanol extracts were added to a final concentration of 1 mg/mL. After incubation for 24 h, growth inhibition of etiologic pathogens KCTC 5527 was evaluated by measuring the absorbance at 600 nm using a spectrophotometer (UV/Vis spectrophotometer UV-1700; Shimadzu Co., Kyoto, Japan) with the same volume of pure methanol as the negative control and expressed as the percent inhibition by each methanol extract.

Considering the ecological niches of the human body (skin and oral cavity), the antimicrobial activities of plant extracts were further determined following the diffusion method using disc paper. Precultured fresh cells (5 × 10^6^ cells/mL) were inoculated onto YPD solid medium following the pour plate method, then dried for 15 min at room temperature. The 6 mm filter paper discs were impregnated with 10 μL of varying concentrations of a plant extract (100 μg/mL, 500 μg/mL, 1 mg/mL, and 2 mg/mL) and then placed on the inoculated agar plates. After cultivation for an appropriate time (24–48 h), the total diameter of the growth inhibition zone, including the paper disc, was measured for each extract concentration. The MIC for each plant extract was defined as the lowest concentration of each tested methanol extract that prevented cell growth (diameter > 8 mm). All experiments were conducted in triplicate to confirm reproducibility.

### 3.5. Determination of the Total Phenolic and Flavonoid Contents

The total phenolic content was determined following the method reported by Folin-Denis et al. [35]. Briefly, 0.2 mL of the methanol extracts (1 mg/mL) was mixed with 0.8 mL of distilled water, 0.5 mL of 2 N Folin–Ciocalteu reagent, and 2.5 mL of 10% NaCO_3_. The resulting mixture was incubated in a water bath at 25 °C for 20 min. To remove precipitates, the reaction mixture was centrifuged at 3000 rpm for 20 min. After centrifugation, the absorbance of the supernatant was measured at 735 nm. The total phenolic content was calculated using a gallic acid calibration curve and expressed in gallic acid equivalents.

The total flavonoid content was determined following the Nieva Moreno method [36]. Briefly, 0.1 mL of the methanol extract (1 mg/mL) was diluted with 0.9 mL of 80% ethanol. Then, 0.5 mL of the resulting mixture was added to a 14 mL test tube containing 4.3 mL of 80% ethanol, 0.1 mL of 10% aluminum nitrate, and 0.1 mL of 1 μM potassium acetate. The resulting solution was incubated at room temperature for 40 min. The absorbance was measured at 415 nm using a spectrophotometer. The total flavonoid content was calculated using a quercetin calibration curve.

### 3.6. Determination of DPPH Antioxidant Activity

The antioxidant activity was measured following the method reported by Abe [37] and Yamaguchi [38] et al. Briefly, 1,1-diphenyl-2-picrylhydrazyl (DPPH; St. Louis, MO, USA) was freshly dissolved in ethanol (95%) to obtain a 0.2 mM DPPH solution. Then, 1.0 mL of the plant extract (1 mg/mL) and 0.1 mL of the DPPH solution were added to a 1.5 mL microtube. After incubation for 10 min in the dark, the changes in color were read at 517 nm using a spectrophotometer (UV/Vis Spectrophotometer UV-1700). A mixture of ethanol (0.1 mL) and plant extract (1 mL) was used as the blank. The control solution was prepared from methanol and the DPPH solution. The scavenging capacity of the antioxidant L-ascorbic acid was used to compare the radical scavenging activity of the plant extracts.

### 3.7. Statistical Analysis

All tests presented in Table 1, Table 2 and Table 3 were independently conducted in triplicates. The results obtained were analyzed using a one-way analysis of variance (ANOVA) followed by Duncan’s multiple range test and reported as the mean ± SD [39].

## Figures and Tables

**Table 1:** Total phenolic and flavonoid contents in plant extracts evaluated in this work.

Botanical Name	Total Phenolic Contents ^a^ (μg/mL)	Flavonoid Contents ^b^ (μg/mL)
*Ilex integra* T. ^1^	140.58 ± 1.11	57.84 ± 1.00
*Osmanthus inularis* K. ^2^	122.52 ± 0.21	19.81 ± 1.34
*Ligustrum obtusifolium* S. and Z. ^3^	71.02 ± 0.27	5.05 ± 0.59
*Eurya emarginata* T. ^4^	119.50 ± 0.86	34.42 ± 1.42
*Camellia japonica* L. ^5^	150.47 ± 1.08	35.20 ± 0.82
*Wasabia koreana* N. ^6^	91.65 ± 0.67	11.88 ± 0.55
*Kirengeshoma koreana* N. ^7^	123.59 ± 1.51	33.38 ± 0.78
*Chrysosplenium flagelliferum* F. ^8^	88.51 ± 1.42	39.74 ± 0.60
*Bistorta manshuriensis* P. ^9^	68.75 ± 0.56	22.89 ± 0.91
*Polygonum sagittatum* var. *sieboldii* L. ^10^	88.07 ± 0.76	23.27 ± 0.54
*Sparganium stoloniferum* B. ^11^	150.16 ± 1.57	31.01 ± 0.62
*Prunella vulgaris* var. *lilacina* L. ^12^	195.96 ± 1.54	34.73 ± 0.89
*Phryma leptostachya* var. *asiatica* H. ^13^	76.12 ± 0.59	20.20 ± 0.12
*Melia azedarach* var. *japonica* M. ^14^	93.06 ± 0.64	47.04 ± 0.45
*Lindera glauca* S. and Z. ^15^	108.70 ± 0.33	27.50 ± 1.45
*Lycoris radiata* L’Her. ^16^	52.43 ± 0.45	12.62 ± 1.51
*Oenothera laciniata* N. ^17^	137.32 ± 0.35	33.47 ± 1.17
*Chelidonium majus* var. *asiaticum* H. ^18^	57.78 ± 1.69	30.01 ± 1.36
*Robinia pseudo-accacia* L. ^19^	139.77 ± 1.56	19.29 ± 1.33
*Eragrostis japonica* T. ^20^	117.22 ± 2.28	15.79 ± 0.87
*Pollia japonica* T. ^21^	39.26 ± 1.37	1.64 ± 0.34
*Hosta minor* N. ^22^	58.43 ± 0.45	9.50 ± 0.31
*Convallaria keiskei* M. ^23^	122.00 ± 0.81	37.81 ± 1.32
*Corylopsis coreana* U. ^24^	150.01 ± 0.14	19.18 ± 0.87
*Carex siderosticta* H. ^25^	139.77 ± 1.83	53.71 ± 0.85
*Hovenia dulcis* T. ^26^	197.12 ± 0.12	62.12 ± 0.12
*Geranium sibiricum* L. ^27^	124.07 ± 0.28	21.25 ± 1.70
*Patrinia scabiosaefolia* F. ^28^	171.30 ± 0.33	51.92 ± 0.93
*Selaginella tamariscina* P. ^29^	50.52 ± 0.99	13.31 ± 0.82
*Firmiana simple* L. ^30^	54.14 ± 1.98	2.43 ± 0.14
*Thuja orientalis* L. ^31^	196.99 ± 1.39	87.99 ± 0.62
*Hedera rhombea* S. and Z. ^32^	91.82 ± 0.69	19.54 ± 0.57
*Aralia continentalis* K. ^33^	78.49 ± 0.41	18.56 ± 1.45
*Aralia elata* A. ^34^	59.28 ± 1.27	23.75 ± 0.45
*Ligularia fischeri* L. ^35^	115.39 ± 0.57	26.51 ± 0.32
*Rosa rugosa* T. ^36^	126.17 ± 0.44	21.85 ± 0.85
*Centella asiatica* L. ^37^	70.18 ± 1.89	28.89 ± 0.27

^1^ Machi tree; ^2^ Island devilwood; ^3^ Privet; ^4^ Emarginate eurya; ^5^ Camellia; ^6^ Wasabi; ^7^ Korean kirengeshoma; ^8^ Stolon golden saxifrage; ^9^ Bistort; ^10^ Arrow-leaf smartweed; ^11^ Bur reed; ^12^ Hagocho; ^13^ Asian lopseed; ^14^ Beed tree; ^15^ Greyblue spicebush; ^16^ Spider lily; ^17^ Evening primrose; ^18^ Asian celandine; ^19^ False acacia; ^20^ Pond lovegrass; ^21^ Japanese pollia; ^22^ Minor hosta; ^23^ Lily of the valley; ^24^ Korean winter hazel; ^25^ Broadleaf sedge; ^26^ Oriental raisin tree; ^27^ Siberian cranesbill; ^28^ Patrinia; ^29^ Selaginella; ^30^ Chinese parasol tree; ^31^ Oriental Arbor vitae; ^32^ Korean ivy; ^33^ Aralia cordata; ^34^ Small-leaf Korean angelica; ^35^ Fischer ligularia; ^36^ Turkestan rose; and ^37^ Asiatic Pennywort. ^a^ Phenolic content was expressed as gallic acid equivalents. ^b^ Flavonoid content was expressed as quercetin equivalents. Values are mean ± SD (n = 3).

**Table 2:** DPPH free radical scavenging activities of plant extracts.

Botanical Name	Free Radical Scavenging Activity ^a^ (% Inhibition)
*Ilex integra* T.	86.96 ± 0.48
*Osmanthus inularis* K.	89.39 ± 0.54
*Ligustrum obtusifolium* S. and Z.	50.49 ± 0.42
*Eurya emarginata* T.	69.41 ± 1.69
*Camellia japonica* L.	92.79 ± 0.31
*Wasabia koreana* N.	39.29 ± 0.53
*Kirengeshoma koreana* N.	74.28 ± 0.58
*Chrysosplenium flagelliferum* F.	68.50 ± 0.92
*Bistorta manshuriensis* P.	55.94 ± 0.27
*Polygonum sagittatum* var. *sieboldii* L.	53.41 ± 0.41
*Sparganium stoloniferum* B.	83.74 ± 1.06
*Prunella vulgaris* var. *lilacina* L.	39.06 ± 0.46
*Phryma leptostachya* var. asiatica H.	61.76 ± 0.50
*Melia azedarach* var. *japonica* M.	31.40 ± 0.57
*Lindera glauca* S. and Z.	81.12 ± 0.80
*Lycoris radiata* L’Her.	48.53 ± 0.54
*Oenothera laciniata* N.	66.50 ± 0.18
*Pollia japonica* T.	17.92 ± 0.74
*Hosta minor* N.	30.72 ± 0.23
*Convallaria keiskei* M.	11.12 ± 0.85
*Corylopsis coreana* U.	73.09 ± 0.98
*Carex siderosticta* H.	42.15 ± 0.15
*Hovenia dulcis* T.	49.59 ± 0.31
*Geranium sibiricum* L.	92.91 ± 0.34
*Patrinia scabiosaefolia* F.	35.22 ± 0.49
*Selaginella tamariscina* P.	46.21 ± 0.17
*Firmiana simple* L.	60.48 ± 1.64
*Thuja orientalis* L.	96.76 ± 0.59
*Hedera rhombea* S. and Z.	15.34 ± 3.14
*Aralia continentalis* K.	14.61 ± 0.53
*Aralia elata* A.	88.60 ± 0.12
*Ligularia fischer* L.	48.62 ± 0.72
*Rosa rugosa* T.	66.55 ± 0.99
*Centella asiatica* L.	89.84 ± 0.50

^a^ Free radical scavenging activity of each sample was determined according to the procedure described in the materials and methods (Section 3.6). Values are mean ± SD (n = 3).

**Table 3:** Antimicrobial activities of plant extracts against *P. acnes* in broth dilution assay.

Botanical Name	Growth Inhibition (%) ^a^
*Ilex integra* T.	93 ± 0.03
*Osmanthus inularis* K.	39 ± 0.91
*Ligustrum obtusifolium S.* and *Z.*	24 ± 1.00
*Eurya emarginata* T.	NO
*Camellia japonica* L.	96 ± 0.70
*Wasabia koreana* N.	52 ± 1.10
*Kirengeshoma koreana* N.	NO
*Chrysospleniumflagelliferum* F.	93 ± 0.40
*Bistorta manshuriensis* P.	33 ± 1.31
*Polygonum sagittatum* var. *sieboldii* L.	91 ± 0.02
*Sparganium stoloniferum* B.	97 ± 0.80
*Prunella vulgaris* var. *lilacina* L.	88 ± 0.19
*Phryma leptostachya* var. *asiatica* H.	54 ± 1.01
*Melia azedarach* var. *japonica* M.	NO
*Lindera glauca* S. and Z.	91 ± 1.03
*Lycoris radiata* L’Her	NO
*Oenothera laciniata* N.	NO
*Chelidonium majus* var. *asiaticum* H.	45 ± 1.00
*Robinia pseudo-accacia* L.	71 ± 0.35
*Eragrostis japonica* T.	NO
*Pollia japonica* T.	40 ± 0.64
*Hosta minor* N.	NO
*Convallaria keiskei* M.	41 ± 0.98
*Corylopsis coreana* U.	NO
*Hovenia dulcis* T.	53 ± 0.27
*Geranium sibiricum* L.	71 ± 1.17
*Patrinia scabiosaefolia* F.	23 ± 0.41
*Selaginella tamariscina* P.	NO
*Firmiana simple* L.	60 ± 1.28
*Thujaorientalis* L.	94 ± 0.68
*Hedera rhombea* S. and Z.	81 ± 1.59
*Aralia continentalis* K.	79 ± 0.57
*Aralia elata* A.	NO
*Ligularia fischeri* L.	NO
*Rosa rugosa* T.	81 ± 0.01
*Centella asiatica* L.	NO

^a^ After the cells were incubated for 24 h, growth inhibition of *P. acnes* was evaluated by measuring the absorbance at 600 nm using a spectrophotometer using methanol alone as the negative control and expressed as percent inhibition by the plant extract. The plant extract was added to a final concentration of 1 mg/mL. Values are mean ± SD (n = 3). NO represents not observed.

**Table 4:** Antimicrobial activities of plant extracts against *P. acnes* in disc diffusion assay.

Botanical Name	Diameters of the Zone of Inhibition (mm)			
	Disc Concentration of a Plant Extract			
	0.1 mg/mL	0.5 mg/mL	1 mg/mL	2 mg/mL
*Ilex integra* T.		8 ^a^	10	10
*Camellia japonica* L.		8	12	12
*Polygonum sagittatum* var. *sieboldii* L.			8	11
*Sparganium stoloniferum* B.			8	10
*Robinia pseudo-accacia* L.			8	10
*Carex siderosticta* H.	8	9	11	13
*Geranium sibiricum* L.	8	11	13	14
*Thuja orientalis* L.	8	8	13	13
*Hedera rhombea* S. and Z.			8	12
*Aralia continentalis* K.	13	18	20	21
*Rosa rugosa* T.			8	10

^a^ The 6 mm filter paper discs were impregnated with 10 μL of varying concentrations of a plant extract (100 μg/mL, 500 μg/mL, 1 mg/mL, and 2 mg/mL) and then placed on the inoculated agar plates. After cultivation for appropriate amounts of time (24–48 h), the total diameter of the growth inhibition zone including the paper disc was measured for each extract concentration. The data represent the mean of three separate experiments.

**Table 5:** List of medicinal plants used in this study.

Botanical Name	Family Name	Korean Name	Plant Parts	Traditional Use
*Ilex integra* T.	Aquifoliaceae	Machi tree	Stem, heartwood	Wounds, simple fractures, toothache
*Osmanthus insularis* K.	Oleaceae	Island devilwood	Stem, bark	Tussis, toothache
*Ligustrum obtusifolium* S. and Z.	Oleaceae	Privet	Privet	Gingival bleeding, halitosis
*Eurya emarginata* T.	Theaceae	Emarginate eurya	Stem	Gingival bleeding, diuretic
*Camellia japonica* L.	Theaceae	Camellia	Leaf	Anti-inflammation, bleeding
*Wasabia koreana* N.	Cruciferae	Wasabi	Whole plant	Antibiotic, dental caries
*Kirengeshoma koreana* N.	Saxifragaceae	Korean kirengeshoma	Root	Food poisoning, toothache, enteritis
*Chrysosplenium flagelliferum* F.	Saxifragaceae	Stolon golden saxifrage	Whole plant	Anti-inflammation
*Bistorta manshuriensis* P.	Polygonaceae	Bistort, snakeweed	Whole plant	Enteritis, bleeding, gingivitis
*Polygonum sagittatum* var. *Sieboldii* L.	Polygonaceae	Arrow-leaf smartweed	Whole plant	Enteritis, antitumor, anti-inflammation
*Sparganium stoloniferum* B.	Sparganiaceae	Bur reed	Whole plant	Wounds, analgesic, melancholia
*Prunella vulgaris* var. *lilacina* L.	Labiatae	Hagocho, selfheal	Whole plant	Fevers, diarrhea, internal bleeding, weaknesses of the liver and heart
*Phryma leptostachya* var. *asiatica* H.	Phrymaceae	Asian lopseed	Whole plant	Detoxification therapy, scabies, anti-inflammation
*Melia azedarach* var. *japonica* M.	Meliaceae	Beed tree, white cedar	Fruit	Vermifuge, scaling
*Lindera glauca* S. and Z.	Lauraceae	Greyblue spicebush	Leaf	Stomach cancer, cancer of the esophagus, toothache
*Lycoris radiate* L’Her	Amaryllidaceae	Spider lily, Red spider lily, cluster amayllis	Leaf	Tonsillitis, antitumor
*Oenothera laciniata* N.	Onagraceae	Evening primrose	Whole plant	Anti-inflammation, dysentery hyperlipidemia
*Chelidonium majus* var. *asiaticum* H.	Papaveraceae	Asian celandine	Whole plant	Wart, atopic dermatitis, anti-inflammation
*Robinia pseudo-accacia* L.	Leguminosae	False acacia	Leaf, stem	Asthma, anti-inflammatory, diuretic
*Eragrostis japonica* T.	Gramineae	Pond lovegrass	Whole plant	Wounds, analgesic
*Pollia japonica* T.	Commelinaceae	Japanese pollia	Whole plant	Wounds, lower back pain, toothache
*Hosta minor* N.	Liliaceae	Minor hosta	Whole plant	Tympanitis, tuberculosis, toothache, gastralgia
*Convallaria keiskei* M.	Liliaceae	Lily of the valley, May lily	Whole plant	Diuretic, edema
*Corylopsis coreana* U.	Hamamelidaceae	Korean winter hazel	Stem	Vomiting
*Carex siderosticta H.*	Cyperaceae	Broadleaf sedge	Whole plant	Headache, toothache, dysmenorrhea
*Hovenia dulcis* T.	Rhamnaceae	Oriental raisin tree	Stem, heartwood	Vomiting, antirheumatic, anti-inflammation
*Geranium sibiricum* L.	Geraniaceae	Siberian cranesbill	Whole plant	Dysentery, detoxification therapy, bleeding, antibiotic
*Patrinia scabiosaefolia* F.	Valerianaceae	Patrinia	Whole plant	Dermatitis, detoxification therapy, dysentery
*Selaginella tamariscina* P.	Selaginellaceae	Selaginella	Whole plant	Bleeding, asthma, nephritis
*Geranium sibiricum* L.	Geraniaceae	Siberian cranesbill	Whole plant	Dysentery, detoxification therapy, bleeding, antibiotic
*Patrinia scabiosaefolia* F.	Valerianaceae	Patrinia	Whole plant	Dermatitis, detoxification therapy, dysentery
*Selaginella tamariscina* P.	Selaginellaceae	Selaginella	Whole plant	Bleeding, asthma, nephritis
*Firmiana simplex* L.	Sterculiaceae	Chinese parasol tree	Stem, bark	Gastralgia, toothache, alopecia
*Thuja orientalis* L.	Cupressaceae	Oriental Arbor vitae, thuja	Stem	Bleeding, colitis, dysentery, insomnia
*Hedera rhombea* S. and Z.	Araliaceae	Korean ivy	Whole plant	Antirheumatic, facial paralysis, antitumor
*Aralia continentalis* K.	Araliaceae	Aralia cordata	Whole plant	Analgesic, diuretic
*Aralia elata* A.	Araliaceae	Small-leaf Korean angelica	Leaf, stem	Anticancer, glycosuria, analgesic
*Ligularia fischeri* L.	Compositae	Fischer ligularia	Whole plant	Asthma, analgesic, vulnerary, pertussis
*Rosa rugosa* T.	Rosaceae	Turkestan rose	Leaf	Bleeding, analgesic
*Centella asiatica* L.	Umbelliferae	Asiatic Pennywort	Whole plant	Anti-inflammation, antitumor, ulcer

## Data Availability

The authors confirm that the data supporting this study are available within the article.
